# Neuroimaging explanations of age-related differences in task performance

**DOI:** 10.3389/fnagi.2014.00046

**Published:** 2014-03-18

**Authors:** Jason Steffener, Daniel Barulli, Christian Habeck, Yaakov Stern

**Affiliations:** ^1^Cognitive Neuroscience Division of the Taub Institute for Research on Alzheimer's Disease and the Aging Brain, Columbia University College of Physicians and SurgeonsNew York, NY, USA; ^2^Department of Neurology, Columbia University of Physicians and SurgeonsNew York, NY, USA; ^3^Department of Psychology, Columbia UniversityNew York, NY, USA; ^4^Department of Psychiatry, Columbia University College of Physicians and SurgeonsNew York, NY, USA

**Keywords:** functional brain activity, cognitive aging, path analysis, moderated-mediation, mediation, task-switching

## Abstract

Advancing age affects both cognitive performance and functional brain activity and interpretation of these effects has led to a variety of conceptual research models without always explicitly linking the two effects. However, to best understand the multifaceted effects of advancing age, age differences in functional brain activity need to be explicitly tied to the cognitive task performance. This work hypothesized that age-related differences in task performance are partially explained by age-related differences in functional brain activity and formally tested these causal relationships. Functional MRI data was from groups of young and old adults engaged in an executive task-switching experiment. Analyses were voxel-wise testing of moderated-mediation and simple mediation statistical path models to determine whether age group, brain activity and their interaction explained task performance in regions demonstrating an effect of age group. Results identified brain regions whose age-related differences in functional brain activity significantly explained age-related differences in task performance. In all identified locations, significant moderated-mediation relationships resulted from increasing brain activity predicting worse (slower) task performance in older but not younger adults. Findings suggest that advancing age links task performance to the level of brain activity. The overall message of this work is that in order to understand the role of functional brain activity on cognitive performance, analysis methods should respect theoretical relationships. Namely, that age affects brain activity and brain activity is related to task performance.

## Introduction

Age has a multifaceted effect on cognitive performance and neural activity. Age-related differences in the neural processes of cognition, investigated with neuroimaging techniques, have led to a variety of explanations and conceptual research models of aging (Cabeza, 2002; Davis et al., 2008; Reuter-Lorenz and Cappell, 2008; Park and Reuter-Lorenz, 2009; Fabiani, 2012). The mechanisms of action of age-related differences in brain activity described in these research models include neural efficiency, capacity and compensation.

Efficiency is the rate at which functional brain activity increases to meet increasing cognitive demands. Therefore, at a given level of cognitive demand someone with greater efficiency requires lower brain activity when compared to someone with lower efficiency. Capacity is the cognitive load at which the maximum amplitude of functional brain activity is reached. Someone with greater capacity has the ability to increase their brain activity over a larger range of cognitive demands then someone with lower capacity. Compensation is functional brain activity in regions not normally utilized to meet cognitive demands. By definition, compensatory functional brain activity is compensating for something. This may simply be a limitation in the functional resources used at lower demands regardless of age, or it may be due to age-related neural changes (Steffener and Stern, 2012). These explanations describe relationships between age-related differences in brain activity as a function cognitive load.

The hemispheric asymmetry in older adults (HAROLD) model describes the increased symmetry in task related functional brain activity with increasing age (Cabeza, 2002). After splitting research participants into high and low performers, this increased symmetry was described as being beneficial and compensatory (Cabeza et al., 2002). The scaffolding theory of aging and cognition (STAC) states that advancing age decreases the efficiency of functional brain networks leading to recruitment of additional brain networks (Park and Reuter-Lorenz, 2009). There is also the idea of compensation-related utilization of neural circuits hypothesis (CRUNCH) (Schneider-Garces et al., 2009). This states that once a neural circuit reaches its capacity with increasing demands it becomes overwhelmed, it then ceases to function effectively and the brain responds with compensatory activity elsewhere in the brain. These theories are extremely important for understanding the aging phenomena. A recent review by Grady (2012) states that a better understanding of age-related neural differences will be gained by explicitly testing relationships between brain activity and task performance (Grady, 2012) This is a key feature of our own research model (Steffener and Stern, 2012) and the focus of the current work.

The goal of the current work was to explain age-related variations in task performance using measures of task-related functional brain activity also affected by advancing age. The hypothesis is that age-related differences in task performance are partially explained by age-related differences in functional brain activity. This assumes that advancing age alters functional brain activity and that there exist a measureable relationship between functional brain activity and task performance, which itself may be affected by advancing age. This approach identifies brain regions whose age-related differences in functional brain activity significantly explain age-related differences in task performance.

Testing this hypothesis used moderated-mediation statistical models. Moderated-mediation analyses describe an analytical framework testing causal relationships between measures, and whether these relationships are dependent on, or interact with, another variable. These are statistical path models where each segment of the path is tested using linear regression. Through combination of the results from each segment, the overall path model is tested and significance is assessed using non-parametric statistics. Moderated-mediation analyses are relatively novel to the neuroimaging field (Wager et al., 2008; Steffener et al., 2011, 2012b); however, are well established in the communications field where they are an active field of research (Preacher et al., 2007; Hayes, 2013).

This analysis approach diverges in subtle but important ways from methods typically used to investigate between group differences in task-related functional brain activity. Standard approaches often test for group differences in brain activity. This is without the constraint that between group activation differences relate to group disparities in performance (Grinband et al., 2008). Other approaches test whether brain activity is predicted by task performance, either controlling for group or testing whether there is a group by performance interaction. These scenarios do not directly identify brain-performance relationships in the presence of group differences in brain activity. Furthermore, standard practices use task-related brain activity as a dependent measure to be explained by task performance. The analyses employed in the current study directly evaluate the theoretical causal model that advancing age affects task-related brain activity, which leads to task performance differences. Additionally, these mediation analyses conform to current views that causal models be employed to test explicit theoretical rationale (Cohen et al., 2003).

The approach used here does not identify all task-related brain regions used by either group, or all task-related brain regions predicting task performance. It identifies only those regions where age-related differences in brain activity directly impact performance. The goals of the current work were addressed using an executive context-switching task (Koechlin et al., 2003; Gazes et al., 2012) administered to a group of younger adults and a group of older adults within the functional MRI environment. This is a task switching experiment requiring cognitive control involving a large number of prefrontal cortical brain regions. Given that advancing age has a major effect on cognitive control resulting in differences in brain activity and performance, it is an ideal task to test for brain-performance relationships.

## Methods

### Study participants

The current study used data from 39 healthy, young participants (18 men and 21 women mean (±*SD*) age = 25.95 (2.92); mean (±*SD*) years of education = 15.64 ± 1.94; all right handed), and 45 healthy, old participants (20 men and 25 women; mean (±*SD*) age = 65.20 ± 2.79; mean (±*SD*) years of education = 15.30 ± 3.08; all right handed). Participants were recruited using a market-mailing approach to equalize the recruitment procedures of young and old. Participants who responded to the mailing were telephone screened to ensure they met basic inclusion criteria (right handed, English speaking, no psychiatric or neurological disorders, normal or corrected-to-normal vision). All participants found eligible via the initial telephone screen were further screened in person with structured medical, neurological, psychiatric, and neuropsychological evaluations to ensure that they had no neurological or psychiatric disease or cognitive impairment. The screening procedure included a detailed interview that excluded individuals with a self-reported history of major or unstable medical illness, significant neurological history (e.g., epilepsy, brain tumor, stroke), history of head trauma with loss of consciousness for greater than 5 min or history of Axis I psychiatric disorder (APA, 1994). Individuals taking psychotropic medications were also excluded. Global cognitive functioning was assessed with the Mattis Dementia Rating Scale, on which a score of at least 133 was required for retention in the study (Mattis, 1988). Informed consent, as approved by the Internal Review Board of the College of Physicians and Surgeons of Columbia University, was obtained prior to study participation, and after the nature and risks of the study were explained. Participants were compensated for their participation in the study.

### Behavioral task

**Figure 1:**
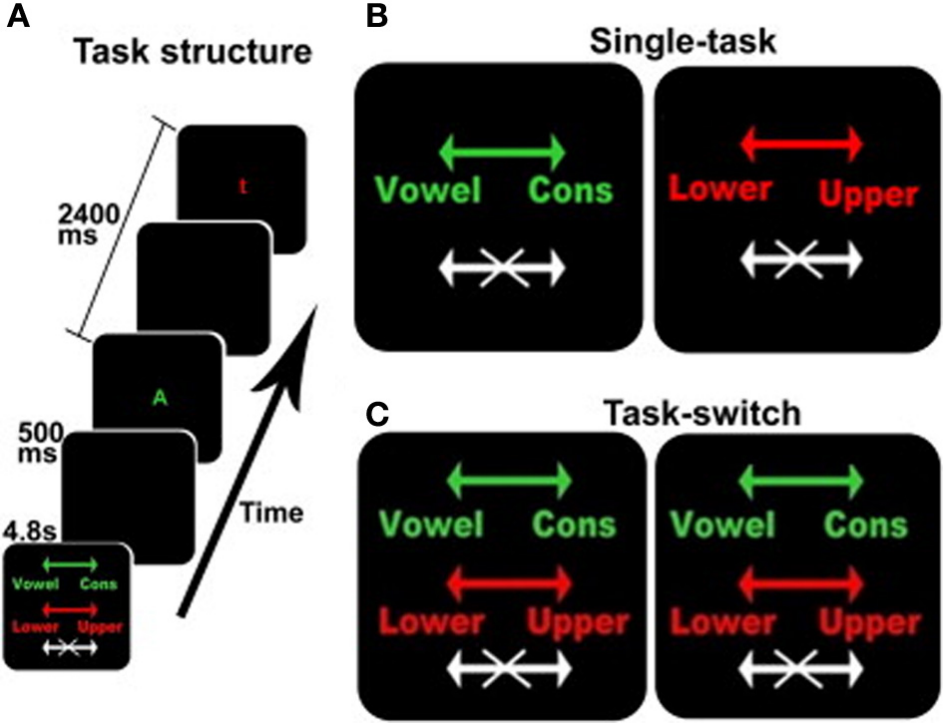
**The switching cognitive task used during fMRI scanning**. **(A)** shows an example of the beginning of a block including the instruction screen for a task-switching block, intertrial interval, a stimulus for the vowel/consonant task, intertrial interval, and a stimulus for the upper-/lower-case task. **(B,C)** show the instruction screens for the single-task and the task-switching conditions, respectively. The colors served as task-cues: green for the vowel/consonant task, red for the upper-/lower-case task, and white for no-go trials. Arrows show the response-hand assignments: left for vowel/right for consonant and left for lower-case/right for upper-case.

The behavioral task was derived from Experiment 2 in the task developed by Koechlin et al. (2003); see Figure 1. This is an intrinsically cued task-switching paradigm with a no-go component where the color of each stimulus served as the task cue. Participants were presented with a series of four conditions comprised of two single-task conditions (Figure 1B) and two identical task-switching conditions (Figure 1C), with the duplication serving to match the number of trials for each discrimination between the single and switch-task conditions (see below). Each block was preceded by a 4.8 s instruction cue to inform the subject of the appropriate action for each stimulus. Each 33.6 s block, comprised 12 sequential letters (or trials) each presented for 1900 ms with an inter-trial time of 500 ms, Figure 1A. Each stimulus was terminated when a response was made or when the trial deadline was reached. These trial dynamics were selected based on performance characteristics of the older adults in behavioral pilot studies, and deviate from Koechlin's briefer presentations (Koechlin et al., 2003). Subjects responded to each letter with a right-hand/left-hand button press or by making no action at all.

In addition to the four active conditions, there were two 33.6 s resting conditions when no stimuli were presented and no response was required. The two resting conditions were identical, but were separately enumerated to simplify description of the Latin Square design (see below). Each resting block presented an instruction cue (“REST”) followed by a blank screen. During fMRI acquisition, each subject was given six repetitions of each of the four active and two resting conditions, for a total of 36 blocks. Conditions were presented in a 6 × 6 fully balanced Latin Square design. The fMRI data acquisition protocol requires stopping the scanner after every six blocks, typically requiring less than 30 s, resulting in the total session duration of approximately 26 min and a total of six fMRI runs with six blocks in each run. Within each of the six runs, all six conditions (two resting, the two single tasks and the two switch tasks) were presented. The rest blocks provided a necessary within task block baseline for assessment of how the amplitude of the fMRI signal changes as a function of task demands. The 6 × 6 Latin square design ensured there were no ordering effects of the different tasks; therefore, although six task blocks were administered, the order of tasks and resting conditions within a block was never the same.

In order to promote the scanning of participants in a stable behavioral and cognitive state, participants were pre-trained on the task and then tested on the entire paradigm in a quiet office prior to the MRI scanning session. Training consisted of giving between one and three blocks of each condition, with unlimited time to inspect the instructions and instruction cues preceding each block, and with auditory feedback indicating incorrect responses. Then participants were tested on the entire 6 × 6 Latin Square identical to the testing protocol described above (pre-scan phase).

### Stimulus presentation

Task stimuli were back-projected onto a screen located at the foot of the MRI bed using an LCD projector. Participants viewed the screen via a mirror system located in the head coil and, if needed, had vision corrected to normal using MR compatible glasses (manufactured by SafeVision, LLC. Webster Groves, MO). Responses were made on a LUMItouch response system (Photon Control Company) using the index fingers. Task administration and collection of RT and accuracy data were controlled using PsyScope 5X B53 (MacWhinney et al., 1997) running on a Macintosh G3/G4 iBook. Task onset was electronically synchronized with the MRI acquisition computer. A MellonIOLabs Systems USB Button Box provided digital input–output for the response system and synchronization with the MRI acquisition computer, as well as millisecond accurate timing of responses.

### MRI data acquisition

MRI images were acquired in a 3.0 T Philips Achieva Magnet using a standard quadrature head coil. A T1-weighted scout image was acquired to determine subject position. One hundred and sixty five contiguous 1 mm coronal T1-weighted images of the whole brain were acquired for each subject with an MPRAGE sequence using the following parameters: TR 6.5, TE 3 ms; flip angle 8°, acquisition matrix 256 × 256 and 240 mm field of view. Six functional scan sets were acquired, each of which included collection of 111 functional images acquired using a field echo echo-planar imaging (FE–EPI) sequence TE/TR = 20/2000 ms; flip angle = 72°; 112 × 112 matrix; in-plane voxel size = 2.0 × 2.0 mm; slice thickness = 3.0 mm (no gap); 41 transverse slices per volume. This functional imaging scan sequence is sensitive to blood oxygen level dependent (BOLD) signal changes. Task induced differences in neural activity alter the ratio of oxygenated to deoxygenated blood in the neurovasculature resulting in altered BOLD signal change which is detectable using this MR scanning sequence. Before the initiation of the executive task, four volumes were acquired and discarded allowing transverse magnetization immediately after radio-frequency excitation to approach its steady-state value. A neuroradiologist reviewed all T1 structural scans with potentially clinically significant findings, such as abnormal neural structure; no clinically significant findings were identified or removed.

### Behavioral analysis

Median response times on correctly answered trials in the single and task-switch conditions were calculated along with the proportion of correctly answered trials under each condition. The response time and accuracy measures were assessed in repeated-measures analysis of variance (ANOVA) with Group (Young/Old) as the between-subject factor and Condition (Single/Task-switch) as the within-subject factors using SPSS (IBM SPSS Statistics for Windows (Version 19.0). Armonk, NY: IBM Corp.). Switch costs were calculated as the difference in the median response times in the task-switch vs. signal task conditions and used as the performance metric with the fMRI data.

### Image pre-processing

All image pre-processing and statistical analyses used SPM8 (Wellcome Department of Cognitive Neurology). For each subject's EPI dataset: images were temporally shifted to correct for slice acquisition order using the first slice acquired in the TR as the reference. All EPI images were corrected for motion by realigning to the first volume of the first session. The T1-weighted (structural) image was co-registered to the first EPI volume using mutual information. This co-registered high-resolution image was used to determine the transformation into a standard space defined by the Montreal Neurologic Institute (MNI) template brain supplied with SPM5. This transformation was applied to the EPI data and re-sliced using sync-interpolation to 2 × 2 × 2 mm. Finally; all images were spatially smoothed with an 8 mm FWHM kernel.

### Time-series analysis

First-level time-series analyses used a block-based model composed of epochs separately representing the single and task-switch conditions. Each epoch was convolved with a canonical model of the hemodynamic response function supplied with SPM8. Contrasts comparing single-task and task-switch conditions were entered into the group-level moderated-mediation analysis.

### Moderated mediation

**Figure 2:**
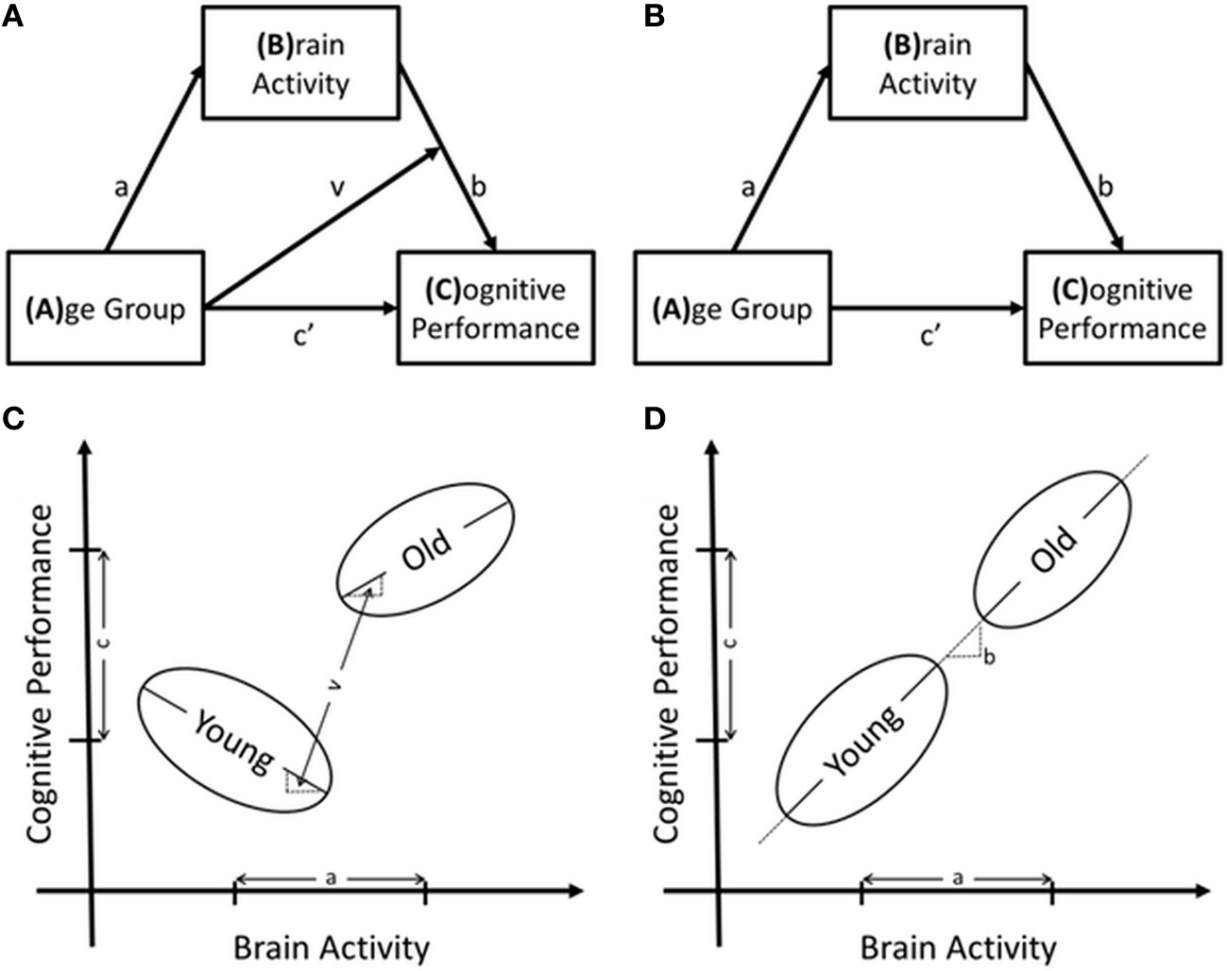
**Structural models of the relationships between age group, brain activity and cognitive performance as well as the potential results to support these models**. In **(A)** is the moderated-mediation model that includes the interaction term and in **(B)** is the mediation model. Regression equations are derived from these models using any box with arrows pointing to it as dependent variables and all variables pointing to the dependent variable as independent variables. The interaction term is included in the regression model as the multiplication of the two independent variables. **(C,D)** are schematics of relationships between the variables that support models A and B, respectively.

This study explored whether task performance was related to the age-related differences in fMRI activity. The first step in this examination identified whether the relationship between age-related differences in brain activity had the same relationship to task performance in both age groups using a moderated-mediation model. This model is shown in Figure 2A and estimates regression Equations 1 and 2a, listed below. The effect of brain activity on task performance in Equation 2a is best demonstrated by rewriting it as equation 2b. The indirect effect of age group on task performance is then calculated by multiplying the effect of age group, “*a*” from Equation 1, and the effect of brain activity, “(*b+vA*)” from Equation 2b. The effect of brain activity on performance is therefore modeled as a function of, or as being conditional on, age group. The indirect effects of age group on task performance are calculated in equation 4. The indirect effect is conditional on age group; therefore, it is calculated for each group, Equations 4b and 4c. Figure 2C, demonstrates an example of a significant moderated-mediation result with reference to the parameter estimates in the model figure and the regression equations.

In the absence of a significant interaction effect, the interaction term was removed, and the moderated-mediation model degenerated to a simple mediation model, Figure 2B. Testing this model used regression equations 1 and 3 and calculated the indirect effect of age-group on task performance through multiplication of the effect of age-group on brain activity, “*a*” from Equation 1, by the effect of brain activity on task performance, “*b*” from Equation 3. To summarize, this model tested whether age-related differences in functional brain activity were directly related to task performance, Figure 2D, demonstrates an example of such a finding.

(1) $$\text{B}=\beta_{0}+a\;\cdot \;A+\epsilon$$

(2a) $$\text{C}=\beta_{0}+b\;\cdot \;B+c^{\prime }\;\cdot \;A+v\;\cdot \;A\;\cdot \;B+\epsilon$$

(2b) $$\text{C}=\beta_{0}+c^{\prime }\;\cdot \;A+\left(b\;+\;v\;\cdot \;A\right)\;\cdot \;B+\epsilon$$

(3) $$\text{C}=\beta_{0}+b\;\cdot \;B+c^{\prime }\;\cdot \;A+\epsilon$$

(4a) Ind=a · (b + v · A)

(4b) $$Ind_{\text{young}}=a\;\cdot \;b|(A=0)$$

(4c) $$Ind_{\text{old}}=a\;\cdot \;b+a\;\cdot \;v|(A=1)$$

The moderated-mediation and simple mediation models were significance tested voxel-wise and all indirect effects were calculated using 5000 age-group stratified bootstrap resamples to determine the bias-corrected percentile confidence intervals (MacKinnon et al., 2004, 2007; Preacher and Hayes, 2008). All analyses used the publically available “Process Models for Neuroimaging” toolbox developed by the author JS (https://github.com/steffejr/ProcessModelsNeuroImage). This is a MatLab toolbox using no additional specialized toolboxes with optional use of “MapReduce” logic for efficient performance across a computational cluster or distributed environment (Dean and Ghemawat, 2008). Age group is a categorical variable and the stratified bootstrapping procedure preserves sample sizes in each age group avoiding bias in the resamples due to the different sample sizes in the age groups. Interpretation of active brain regions for the moderated-mediation and mediation effects used voxel-wise height thresholds of *p* < 0.05 and cluster extent of 50 suprathreshold contiguous voxels.

## Results

### Behavioral results

Performance decreased in the task-switch vs. single task condition and with advancing age for both median response time and the proportion of correct trials. There was a significant interaction between age group and task condition (task-switch vs. single) for both median response time (RT) [young: mean (std) = 0.22 (0.093), old: mean (std)=0.29 (0.16), *F*_(1, 82)_ = 4.72, *p* = 0.033] and proportion correct [young: mean(std) = −0.025(0.043), old: mean(std) = −0.070(0.066), *F*_(1, 82)_ = 13.07, *p* = 0.001]. Difference scores were calculated between task-switch and single tasks and there were age group effects for both median RT [*t*_(72.10)_ = −2.25, *p* = 0.027] and proportion correct [*t*_(76.01)_ = 3.73, *p* < 0.001]. Means and standard deviations are listed in Table 1.

**Table 1:** **Demographics and performance values**.

	**Young**		**Old**	
	**Mean**	**Std**	**Mean**	**Std**
Age	25.95	2.92	65.20	2.79
Education	15.64	1.94	15.30	3.08
DRS	140.74	2.39	140.00	3.13
Single, median RT	0.690	0.079	0.820	0.140
Dual, median RT	0.970	0.120	1.110	0.230
Single, proportion correct	0.940	0.097	0.900	0.120
Dual, proportion correct	0.920	0.110	0.830	0.130
Dual—single, median RT	0.220	0.093	0.290	0.160
Dual—single, proportion correct	−0.025	0.043	−0.070	0.066

### Brain imaging results

First presented are moderated-mediation results, followed by mediation results in locations not demonstrating a significant moderated effect. Results are presented with brain overlays and tables, data from key loci are presented in scatter plots to aid in the interpretation. Moderated-mediation results all require a significant interaction effect between age group and brain activity in regions demonstrating age-related effects in predicting task performance. Mediation results demonstrate age-group differences in brain activity that predict task performance in brain regions having a non-significant interaction effect. Therefore, results from these two tests are exclusive of each other.

#### Moderated-mediation

**Figure 3:**
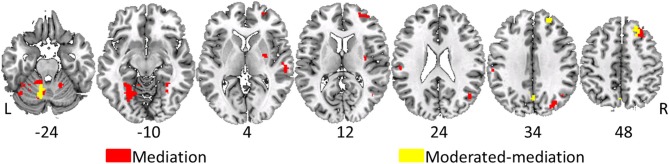
**Overlay of voxels having significant moderated-mediation effects of age-group on task performance via fMRI activity in yellow**. Voxels having significant mediating effects are in red. Voxel designated as having moderated-mediation effects had significant interaction effects between age group and fMRI activity in predicting performance and a significant indirect effect. Significant mediating effects require a non-significant interaction effect with a significant indirect effect. Significance assessed at *p* < 0.05 and indirect effects assessed with 5000 bias-corrected, accelerated, and stratified bootstrap resamples. A cluster extent of 50 contiguous voxels minimized false positives. Left is on the left in these images and the numbers under the slices indicate z-plane location in millimeters of the MNI template space.

Without exception, significant moderated-mediation effects of age group on performance via brain activity was driven by the older age group via significant relationships between increasing brain activity and decreasing task performance; however, there was no relationship between increased activation and performance in the young group. Clusters of voxels demonstrating significant moderated-mediation effects between age group and age-related decreases in task performance (increased switch costs) are listed in Table 2 and shown as overlays in Figure 3. Only clusters exceeding 50 voxels in size where the interaction effect was significant at *p* < 0.05 and the indirect effect was significant in at least one age-group are considered to support a moderated-mediation finding. Table 2 includes parameter estimates *a* representing the size and significance of the between age group effects in brain activity. Parameter *b* represents the size of the main effect relationship between brain activity and task performance accounting for age group. Parameter *c*' is the size of the main effect of age group on task performance after accounting for brain activity. Parameter *v* is the interaction effect size between age group and brain activity. This parameter is also interpretable as the difference between the within group brain activity to performance relationships. Between group effects, parameter *a*, can result for multiple reasons; therefore, within group brain activity measures are included in Table 2 for the dual > single condition. The indirect effects in Table 2 were calculated using Equations 4b and 4c.

**Table 2:** **Moderated-mediation analysis results**.

**Region**	**Lat**	**BA**	***x***	***y***	***z***	**Cluster**	***a***	***b***	***c*′**	***v***	**Dual > single**		**Indirect effects**	
						**size**					**Young**	**Old**	**Young**	**Old**
Sup. frontal	R	9	20	32	52	77	0.625^*^	−0.021	0.041	0.109^*^	−0.451	0.174	−0.013	0.055^*^
Mid. frontal	R	–	32	26	50	–	0.542^*^	0.004	0.045	0.053^*^	0.261	0.804^*^	0.002	0.031^*^
Mid. frontal	R	–	22	48	34	54	−0.516	−0.006	0.076^*^	0.066^*^	0.406^*^	−0.111	0.003	−0.031^*^
–	–	–	−8	−54	−26	87	0.441^*^	−0.010	0.049	0.089^*^	−0.001	0.440^*^	−0.004	0.035^*^
Cerebellum	L	18	−10	−70	−22	–	0.543	0.009	0.04	0.049^*^	0.205	0.748^*^	0.005	0.032^*^
Precuneus	L	7	−4	−64	42	120	0.666^*^	−0.002	0.044	0.048^*^	0.646^*^	1.312^*^	−0.001	0.031^*^

*Regions included are those with significant moderated-mediation effects where the effect of age-related differences in functional brain activity on task performance differed between the age groups. In order to meet this criterion a region needs to have a significant interaction effect, v and have a significant indirect effect for at least one age group. Significance of the parameter estimates and the indirect effects are tested at p < 0.05 and indicated with a star. The “Dual > Single” column shows the within group measures and significance, as identified with a star, indicates whether the within group effect differs from zero. These values aid in the interpretation of the parameter estimate a, which tests the between group effects. Height threshold of p < 0.05 and cluster size > 50. BA, Brodmann Areas; – refers to local maxima or locations without atlas labels. Values refer to unstandardized parameter estimates*.

**Figure 4:**
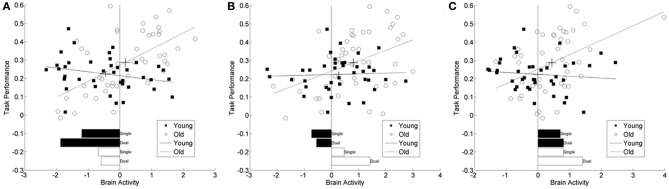
**Scatter plots from three representative brain locations. (A)** superior frontal gyrus (20, 32, 52), **(B)** middle frontal gyrus (22, 48, 34), **(C)** cerebellum (−5, −54, −26). Each panel plots brain activity vs. task performance within age group, greater numbers reflect greater switch costs or worse performance. The lines correspond to the regression fit lines within each group and the cross-hairs on these lines are located at the mean values for brain activity and task performance. The length of the cross hairs corresponds to the standard errors of the means. Young adults are represented with filled in circles and solid fit lines and old adults with open circles and dashed fit lines. Below the scatter plots are bar plots for the simple within group, with task demand bar plots in the same units as the scatter plots. These values are comparable to the *t*-values listed in Tables 2, 3 and aid in the interpretation of these results. In **(C)** there appears to be an outlier in the brain activity measure for one older adult. Excluding this participant and repeating the analysis for this location does not change the significant results.

To aid in the interpretation of these results scatterplots plots of a selection of loci are shown in Figure 4. In all but one location, the difference in brain activity between the two task conditions was greater in the older then the younger adults. Although the moderation-mediation results are similar across regions, the underlying within group differences in brain activity leading to these findings differed across regions. As an example, some regions showed brain activity in the positive direction while others in the negative direction. Therefore, careful consideration of the results is required for accurate interpretation. Several locations discussed in detail interpret various exemplars of results.

Inspection of results from the right superior frontal cortex (BA 9) had a significant decrease in fMRI signal, relative to baseline, for both task conditions in the young group, see bar plot in Figure 4A. The old group however, had decreased activity to a lesser degree. Within the middle frontal cortical location, the young group also had decreased activity, relative to baseline, while the older group had increasing activity with increasing task demands, Figure 4B. A separate inferiorly located cluster, also within the right middle frontal cortex, had more decreased activity in the young group then the old age group. The cluster within the left cerebellum had activity in the positive direction for all task demands and both age groups; however, there was a significant increase in activity with increasing task demands for the old age group, Figure 4C. The cluster in the precuneus (BA 7) only demonstrated significant activity in the old age group at high task demands. In each of these locations, the task-related signal change differed between the age groups and the relationship of this activity to task performance differed by age group.

#### Mediation results

After masking out locations with significant moderated-mediation effects, clusters of contiguous voxels all demonstrating significant mediation effects between age group and decreases in task performance (increased switch costs) were identified and are listed in Table 3. The locations in the right superior temporal cortex (BA 21/22) and the right putamen each had decreased activity in the task-switch vs. single task condition for the young group; the old group also had decreased activity, but to a lesser degree then the young adults. The third cluster, including the left cerebellum and extending into the fusiform gyrus, had consistent responses throughout both levels of task demands and age groups. The young adults had non-distinguishable high levels of activity for both levels of task demands while the older adults had significantly larger increases in activity with increasing demands. The fourth cluster, including areas of the right middle temporal into occipital cortices, had significant suppression of activity for the young group; however, the old age group did not have significant activity in either direction. Within the fourth cluster the task-related activity differs between the local maxima. The first maxima within right middle frontal gyrus (BA 46) has increasing task-related activity with increasing task demands for the young age group; however, the old age group has significant activity for both levels of demand but less for the greater cognitive load. There was no significant task-related activation for either level of task-demand, nor did age group in the second local maxima of this cluster and in the third, only the young group at low task demands have significant suppression of activity. The next two clusters of cerebellar activity had similar behavior as the previous cerebellar finding.

**Table 3:** **Mediation analysis results**.

**Region**	**Lat**	**BA**	***x***	***y***	***z***	**Cluster**	***a***	***b***	***c'***	**Dual > single**	
						**size**				**Young**	**Old**
Sup. temporal	R	21	60	−28	2	81	0.809^*^	0.044^*^	0.028	−0.686	0.123
Sup. temporal	R	22	54	−18	2	–	0.452^*^	0.035^*^	0.047	−0.401	0.052
Putamen	R	48	32	−8	10	52	0.606^*^	−0.040^*^	0.087^*^	−0.499	0.108
–	–	–	−10	−64	−28	624	0.698^*^	0.036^*^	0.038	−0.011	0.687^*^
Cerebellum	L	18	−10	−74	−20	–	0.538	0.044	0.040	0.159	0.696^*^
Fusiform	L	19	−32	−76	−16	–	0.652^*^	0.036^*^	0.040	0.272	0.924^*^
Mid. occipital	R	39	46	−66	26	78	0.707^*^	0.036^*^	0.038	−0.254	0.453^*^
Mid. temporal	R	37	44	−62	12	–	0.639^*^	0.023^*^	0.049	−0.598	0.041
Mid. frontal	R	46	38	52	12	78	−1.010	0.023	0.086^*^	0.716^*^	−0.293
Mid. frontal	R	10	26	56	6	–	−0.664	0.030	0.083^*^	0.553^*^	−0.110
Sup. frontal	R	10	22	58	14	–	−0.775	0.021	0.079^*^	0.556^*^	−0.219
Cerebellum	L	–	−42	−64	−28	160	0.630^*^	0.036^*^	0.041	0.168	0.799^*^
Cerebellum	L	37	−30	−48	−28	–	0.629^*^	0.033^*^	0.042	−0.111	0.518^*^
Cerebellum	R	37	16	−50	−24	56	0.670^*^	0.034^*^	0.040	−0.060	0.610^*^
Sup. occipital	R	19	26	−80	34	71	0.739^*^	0.031^*^	0.041	−0.279	0.460^*^
Mid. frontal	R	9	30	28	48	59	0.573^*^	0.038^*^	0.041	−0.003	0.571^*^
Sup. frontal	R	9	24	36	46	–	0.595^*^	0.025^*^	0.049	−0.283	0.312
Inf. parietal	L	2	−52	−26	38	58	0.560^*^	0.039^*^	0.042	−0.502	0.059
SupraMarginal	L	48	−54	−22	24	–	0.500^*^	0.037^*^	0.045	−0.532	−0.032
Fusiform	R	37	30	−48	−14	71	0.573^*^	0.037^*^	0.042	−0.152	0.422^*^
Fusiform	R	37	30	−56	−12	–	0.528^*^	0.027^*^	0.049	0.021	0.550^*^
–	–	–	−10	−48	−26	106	0.461^*^	0.044^*^	0.043	−0.061	0.401^*^
Cerebellum	L	18	−4	−48	−14	–	0.386	0.036	0.049	0.079	0.465^*^
^**^Empty^**^	–	–	−2	−34	−26	–	0.425	0.033	0.050	0.124	0.548^*^

*Regions included are those with significant mediation effects where the effect of age-related differences in functional brain activity on task performance differed between the age groups. In order to meet this criterion a region needs to have a significant interaction effect, v and have a significant indirect effect for at least one age group. Significance of the parameter estimates are tested at p < 0.05 and indicated with a star. The “Dual > Single” column shows the within group measures and significance, as identified with a start, indicates whether the within group effect differs from zero. These values aid in the interpretation of the parameter estimate a, which tests the between group effects. Height threshold of p < 0.05 and cluster size > 50. BA, Brodmann Areas, – refers to local maxima or locations without atlas labels. Values refer to unstandardized parameter estimates*.

## Discussion

The present study investigated how age-related effects on task performance are partially explained by measured age-related differences in brain activity. Results from two statistical models: moderated-mediation and simple mediation indicated that across the brain, there exist different relationships between age, brain activity, and cognitive task performance. Interestingly, certain aspects of the results were very similar across the two models. In all identified locations, significant moderated-mediation relationships resulted from increasing brain activity predicting worse (slower) task performance in older but not younger adults. Therefore, findings from this study suggest that advancing age links task performance to the level of brain activity. One explanation is that the task is not sufficiently demanding enough in the younger adults for performance to be dependent on measurable brain activity. This concept is supported by the thesis of Fabiani, that advancing age moves participants along a continuum relationship between neural activity and task performance without altering the relationship (Fabiani, 2012). It is important however, to keep in mind the nature and assumptions of the current analyses. The current models test the hypothesis that age's effect on task performance is mediated by brain activity. The aim of this work was not to identify brain regions having differential activity as a function of age group or brain regions with activity that predicts performance. The aim was to address the question of whether age-related neural differences account for age-related differences in task performance.

From our own results, regions of the right pre-frontal cortex were identified from the moderated-mediation and simple mediation results. Investigation of the within group simple effects show differential directions of activity within the cluster, see Figures 4A,B. Based on very simple interpretations and assumed accuracy in foci identification, the distinction is between the middle and superior frontal gyri. The middle frontal areas demonstrated that in the older adults, increased activation predicted worse performance. This suggests an efficiency interpretation whereby activity increases with increased task demands leading to slower task performance. Within the superior frontal area of this cluster, suppression of activity with increasing task demands was related to worsening performance. These different directions of responses, within the same clusters, demonstrate the complex relationships between brain activity and performance. These findings suggest that frontal regions are mediating the effect of age on activation differentially depending on their location and the task load. This result should not be surprising given the theoretical importance placed on frontal regions to various compensation theories of cognitive aging. As certain posterior regions deteriorate, frontal regions are called upon to reorganize and to functionally compensate (Davis et al., 2008), leading to nuanced patterns depending on the manner of that reorganization. While these results on their own do not shed light on the specific patterns of reorganization we may expect to find, they do rule out simplistic interpretations of frontal reorganization.

Our findings within in the prefrontal regions are encouraging and support the work by the Koechlin et al. (2003) who developed the task used (Koechlin et al., 2003). Subsequent work, with a similar task, also found PFC activity along with posterior-parietal (Jimura and Braver, 2010), similar to our own findings within the PFC and posterior-parietal regions. Notably, this work identified sustained and transient task-related signal change within the many portions of the PFC. Within the anterior PFC these authors identified that the relationship between increasing transient brain activity and increased switch costs was greater in the old than the young. Our experiment only investigated sustained activity across blocks of trials; however, our results support this PFC finding by Jimura and Braver (2010). The experiment by Madden et al. (2010) also investigated transient activity comparing switch trials to non-switch trials to identify frontoparietal network of brain activity (Madden et al., 2010). These authors continued their work to identify age-related differences in functional connectivity between switch-task related brain regions. This avenue of exploration is a potential future direction of our own work.

Other brain regions identified in these analyses included the cerebellum. Previous findings suggest that the cerebellum is especially important for task switch processing (Wu et al., 2013). Here we found that the left cerebellum showed high levels of activation across task loads for both young and old subjects, but especially high levels of activation for older subjects under high loads. The frontal regions, previously discussed, are consistent with many theories of frontal compensatory activation; however, the cerebellum appears more involved with the integration of motor and cognitive networks necessary to perform task switching. Hence, greater activation in this region among the older adults at the highest level of load suggests a relatively straightforward efficiency interpretation; whereas young subjects can integrate such networks effectively with the same levels of activation across task loads. Alternatively, older subjects must increase their cerebellar activation at the highest levels of load to do so effectively.

### Variance accounted for

In order to put these results into some larger perspective it is important to point out that age group alone accounted for 5.4 percent of the variance in switch costs; although significant this is a relatively small amount of the total variance. Using age group and brain measures at all voxels to account for as much variance as possible in task performance, 32.7 percent of total the variance in switch costs was accounted for. This increase of 27 percentage points demonstrates the value of including measures of brain activity in attempting to explain age group differences in task performance. Some of the remaining variance in task performance may be attributable to differences the grey matter and cerebral blood flow (Steffener et al., 2012a).

### Compare process models to single model regression analyses

One important distinction about the approach implemented here is how and why the presented results may differ from similar analyses. The current analyses are process models (moderated-mediation and simple mediation) meaning they test for pathways of effects. This is in contrast to regression models that predict brain activity using task performance, either interacting with age group or controlling for age group. The difference is that the process models test the hypothetical causal pathways and ensure the presence of age group differences in brain activity. Therefore, there needs to be an effect of age group on brain activity in order to test for relationships between brain activity and task performance. In the absence of this restriction, interesting brain-behavior relationships may be identified; however, the focus of the current work was on explaining how age differences in brain activity explain task performance.

Another difference is that the process models order the testing of regression equations to reflect the assumed causal relationships between the variables. As an example, we can discuss the brain to performance branch of the process model, Equation 2 above. Within the framework of standard neuroimaging software packages (e.g., SPM and FSL) this regression equation is restructured as follows with the brain measure as the dependent variable:

(5) B=gA+hC+j · A · C+ϵ

This formulation does not reflect the assumed causal nature of effects taken in the current work: that advancing age affects brain activity and that brain activity affects task performance. The causal nature of regression Equation 5 is that task performance and age group jointly affect brain activity. While this may simply seem to be semantics, there is a real difference in the statistical results. As a simple demonstration, the data from the first two cluster maxima having significant moderated-mediation results were analyzed with Equations 2 and 5. The interaction effect *t*-value from the location in the superior frontal gyrus region (20, 32, 52) is 4.74 using the causal model (Equation 2) and 3.62 using equation 5. The interaction effect *t*-value from the other location in the middle frontal gyrus region (22, 48, 34) is 3.03 using the causal model (Equation 2) and 1.90 using Equation 5. Therefore, using the regression equation formatted in standard software packages does not test theoretical causal concepts, and it has different statistical results that may not identify the intended relationships under study.

### Limitations

One caution in the interpretation of the results is that a low statistical height threshold was used. Use of a relatively large cluster extent threshold compensated for this; however, there is still the possibility of false positives. Additionally, standard normalization templates were used for registering all brain images into a common space. This may result in acceptable but less than ideal spatial transformations.

Interpretations of these results suggest areas compensating for decreased efficiency and capacity of other brain regions. None of the analyses of the current work addresses the suggestion that brain activity in one region is due to alterations in another region. In previous work, we found brain-imaging evidence to support the idea of compensation, but cautioned that this interpretation can only be conclusive upon identification of what brain area in one location is compensating for (Zarahn et al., 2007). Follow-up analyses linked increased brain activity in the older adults to decreased gray matter volume (Steffener et al., 2009). The idea however, that increased activity in one area may be compensating for decreased activity in another, implies multivariate covariance analyses which use the entire brain to identify patterns of interacting brain activity (Habeck et al., 2003), and these patterns themselves may also interact with each other (Steffener et al., 2012b). Therefore, the current results require follow-up investigation in–line with our conceptual research model to put them in perspective and to offer a more general understanding of the aging brain (Steffener and Stern, 2012).

## Conclusions

The overall message of this work is that the current approach formulates the analysis with respect to theoretical considerations. Namely, that age affects brain activity and brain activity is related to task performance. The current findings identified brain regions whose age-related differences in functional brain activity significantly explained age-related differences in task performance. In all identified locations, significant moderated-mediation relationships resulted from increasing brain activity predicting worse (slower) task performance in older but not younger adults. Findings suggest that advancing age links task performance to the level of brain activity. The computational burden from the current approach is greater than standard methods; however, the result may be interpreted with direct relation to hypotheses. The current approach also represents a small step toward complete integration of multiple modalities of brain images, cognitive performance and moderating variables to better understand cognitive aging.

### Conflict of interest statement

The authors declare that the research was conducted in the absence of any commercial or financial relationships that could be construed as a potential conflict of interest.
